# Stable nuclear transformation of *Eudorina elegans*

**DOI:** 10.1186/1472-6750-13-11

**Published:** 2013-02-12

**Authors:** Kai Lerche, Armin Hallmann

**Affiliations:** 1 Department of Cellular and Developmental Biology of Plants, University of Bielefeld, Universitätsstr. 25, D-33615, Bielefeld, Germany

**Keywords:** Co-transformation, *Gaussia princeps* luciferase gene, Genetic engineering, Green algae, Heterologous expression, Reporter genes, Selectable markers, *Streptomyces rimosus aph*VIII gene, Volvocaceae, Volvocine algae

## Abstract

**Background:**

A fundamental step in evolution was the transition from unicellular to differentiated, multicellular organisms. Volvocine algae have been used for several decades as a model lineage to investigate the evolutionary aspects of multicellularity and cellular differentiation. There are two well-studied volvocine species, a unicellular alga (*Chlamydomonas reinhardtii*) and a multicellular alga with differentiated cell types (*Volvox carteri*). Species with intermediate characteristics also exist, which blur the boundaries between unicellularity and differentiated multicellularity. These species include the globular alga *Eudorina elegans*, which is composed of 16–32 cells. However, detailed molecular analyses of *E. elegans* require genetic manipulation. Unfortunately, genetic engineering has not yet been established for *Eudorina*, and only limited DNA and/or protein sequence information is available.

**Results:**

Here, we describe the stable nuclear transformation of *E. elegans* by particle bombardment using both a chimeric selectable marker and reporter genes from different heterologous sources. Transgenic algae resistant to paromomycin were achieved using the aminoglycoside 3^′^-phosphotransferase VIII (*aph*VIII) gene of *Streptomyces rimosus*, an actinobacterium, under the control of an artificial promoter consisting of two *V. carteri* promoters in tandem. Transformants exhibited an increase in resistance to paromomycin by up to 333-fold. Co-transformation with non-selectable plasmids was achieved with a rate of 50 - 100%. The luciferase (*gluc*) gene from the marine copepod *Gaussia princeps*, which previously was engineered to match the codon usage of *C. reinhardtii*, was used as a reporter gene. The expression of *gluc* was mediated by promoters from *C. reinhardtii* and *V. carteri*. Heterologous heat shock promoters induced an increase in luciferase activity (up to 600-fold) at elevated temperatures. Long-term stability and both constitutive and inducible expression of the co-bombarded *gluc* gene was demonstrated by transcription analysis and bioluminescence assays.

**Conclusions:**

Heterologous flanking sequences, including promoters, work in *E. elegans* and permit both constitutive and inducible expression of heterologous genes. Stable nuclear transformation of *E. elegans* is now routine. Thus, we show that genetic engineering of a species is possible even without the resources of endogenous genes and promoters.

## Background

The evolution of multicellularity and the separation of germline cells from sterile somatic cells are certainly among the greatest innovations of eukaryotes. Remarkably, phylogenetic analysis suggests that the shift from unicellular to multicellular organisms with differentiated cells was not a unique progression in the evolution of life, but it was, in fact, a frequent event [1-4].

Particularly with regard to questions related to the evolution of multicellularity and of the germ-soma dichotomy, the volvocine green algae within the Volvocales (Chlorophyta) order are of special interest. The volvocine green algae form a closely related group of flagellated, photosynthetic, phototactic, facultatively sexual haploid eukaryotes which include unicellular, colonial and multicellular forms with differentiated cell types [5-7]. Several genera of the volvocine lineage can be arranged in a conceptual series according to increasing morphologic and developmental complexity (from *Chlamydomonas* through *Gonium*, *Pandorina*, *Eudorina* and *Pleodorina* to *Volvox*) [5-7]. In this series there are progressive increases in cell number, organismal polarity, volume of extracellular matrix per cell, size of adult organisms, and the tendency to produce sterile, terminally differentiated somatic cells [5-7]. The unicellular alga *Chlamydomonas* shows the simplest possible morphologic and developmental complexity in this conceptual series; the cell grows in size and cell division produces unicellular daughter cells. The colonial alga *Gonium* has the shape of a slightly convex plate, typically containing 8-16-cells; all cells grow and divide to produce new colonies. *Pandorina* forms a 16-celled ellipsoid colony; all its cells grow and divide. *Eudorina* is a 16–32 celled, globular alga; generally, all cells of *Eudorina* grow and divide, but in some instances the 2–4 most anterior cells fail to divide and act as somatic cells [7,8]. *Pleodorina* is a 32–128 celled, globular alga, in which all anterior cells remain small and function as non-dividing somatic cells, while all posterior cells grow and divide. The multicellular, globular alga *Volvox* exhibits the most advanced morphologic and developmental complexity in the volvocine lineage; it consists of many hundreds to thousands of cells and almost all cells terminally differentiate as small, biflagellate somatic cells, while only a very few cells grow up to many times their initial size and then divide to produce new individuals.

The diversity of volvocine forms provides an ideal model system for addressing fundamental molecular issues related to the transition to differentiated multicellularity. So far, the most intensively studied members of the volvocine algae group with sequenced genomes and well-established molecular-genetic tools are *Chlamydomonas reinhardtii*[9,10] and *Volvox carteri*[4-7]. Less research has been done into the molecular characteristics of volvocine genera with intermediate organizational complexity between unicellular forms and multicellular forms with fully differentiated somatic and germ cells; these genera include *Gonium*[7,11-17], *Pandorina*[7,18-23], *Eudorina*[7,8,24-36] and *Pleodorina*[7,13,37-43].

**Figure 1:**
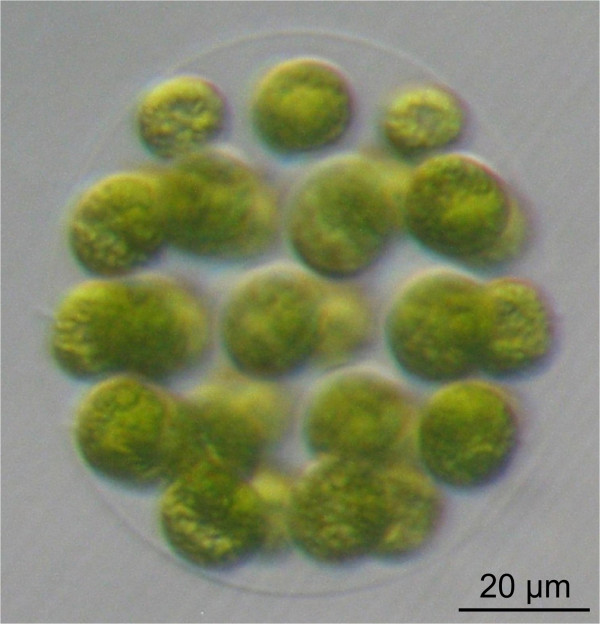
**The wild**-**type phenotype of *E. elegans.*** A vegetatively grown spheroid of *E*. *elegans* with ~32 biflagellate cells at the surface of the organism is shown. Each round-shaped cell contains a single large, cup-shaped chloroplast. Most of the spheroid volume consists of a complex but transparent glycoprotein-rich extracellular matrix that holds all of the cells in place.

Detailed molecular analyses of volvocine genera with intermediate organizational complexity require genetic manipulation. Therefore, our goal was to establish the stable, nuclear transformation of *E. elegans*, a volvocine species with intermediate organizational complexity (Figure 1). Molecular-genetic tools do currently not exist for *E. elegans* and this species has not been genetically engineered previously. We intended to develop a biolistic transformation method because previous biolistic methods were successful in three volvocine species, *V. carteri*[44], *G. pectorale*[17], and *C. reinhardtii*[45]. It should be mentioned that transformation methods using glass beads [46] or electroporation [47] are inappropriate for *Eudorina* because they require cell-wall deficient target cells, which do not exist for this species. Moreover, only limited sequence information is available for *Eudorina*, with the exception of several short DNA fragments that have been used as phylogenetic marker sequences [40,48-58].

An important prerequisite for transformation is a selectable marker gene for the identification of putative transformants. Under the given conditions of *Eudorina*, a heterologous, dominant marker was preferred because it allows for the transformation of wild-type strains, i.e., auxotrophic mutants are not required.

Transformation protocols using the heterologous phleomycin-resistance (*ble*) gene of *Streptoalloteichus hindustanus*[59-61] as a dominant selectable marker have been established for *C. reinhardtii*[62] and *V. carteri*[63]. The aminoglycoside 3^′^-phosphotransferase VIII (*aph*VIII) gene of *Streptomyces rimosus*[64] has been used as a dominant marker in *C. reinhardtii*[65,66], *G. pectorale*[17], and *V. carteri*[66,67]. Additional dominant selectable markers have only been established in *C. reinhardtii*. The corresponding protocols utilize the R100.1 plasmid/bacteriophage T4/synthetic aminoglycoside adenyltransferase (*aadA*) gene [68], the *Streptomyces hygroscopicus* aminoglycoside phosphotransferase (*aph7”*) gene [69], the mutated version of the protoporphyrinogen oxidase (*PPX1*) gene [70], the *C. reinhardtii* acetolactase synthase (*ALS*) gene [71], and the mutated version of the *C. reinhardtii* ribosomal protein gene S14 (*CRY1*) [72].

Among the dominant selectable markers described above, only the *aph*VIII gene [17,65-67] and the *ble* gene [62,63] have been used successfully for the transformation of more than one volvocine algae species. We decided to use the *aph*VIII gene because it was successfully used in three volvocine species. Furthermore, the *aph*VIII gene encodes an enzyme and not simply a binding protein like Ble, which requires a 1:1 ratio of expressed protein and antibiotic molecules. A heterologously expressed enzyme should allow for selection even in the event of a low expression rate.

Selectable marker genes need to be expressed homogeneously in transgenic organisms using (strong) promoters. Therefore, established molecular tools for volvocine algae routinely utilize promoters of highly expressed endogenous genes. For example, the promoters of the ribulose bisphosphate carboxylase small chain (*RBCS2*) gene [62], the *PSAD* gene of photosystem I [73], a fused promoter containing part of the heat shock protein 70A (*HSP70A*) promoter and part of the *RBCS2* promoter [74], and another fused promoter containing part of the heat shock protein 70A (*HSP70A*) promoter and part of the β_2_-tubulin promoter (*β*_*2*_*TUB*) [74] have been utilized in *C. reinhardtii.* For *V. carteri*, the β-tubulin promoter [75], the promoter of the arylsulfatase (*ars*) gene [76,77], and a *hsp*70A/*rbc*S3 fusion promoter [67] have been established. Because *E. elegans* has not been sequenced and because there were no endogenous promoter sequences available, we intended to test heterologous promoters. Though promoter sequences are frequently species-specific, it has been shown that some promoters also work in related volvocine species [17,66].

Research with transgenic organisms also requires reporter genes. Genes encoding the green fluorescent protein (*gfp*) of *Aequorea aequorea*[78], the arylsulfatase (*ars*) gene of *V. carteri*[77], the hexose/H^+^ symporter (*hup*1) gene of *Chlorella kessleri*[75], and the luciferases of *Renilla reniformis* (*rluc*) [79,80] and *Gaussia princeps* (*gluc*) [17,81,82] have been used successfully in volvocine algae (mainly *C. reinhardtii* and *V. carteri*). For experiments in *Eudorina*, a codon-adapted version of the *G. princeps* luciferase gene seemed suitable because luciferase exhibits a very high enzymatic activity even when expressed at low levels [82]. Additionally, luciferase can be used for large-scale screening by a simple enzymatic assay, and it has been shown to work in two volvocine species, *C. reinhardtii*[82] and *G. pectorale*[17].

Here, we demonstrate the stable nuclear transformation of *E. elegans* by particle bombardment using a chimeric, *aph*VIII-based selectable marker gene driven by a tandem promoter of *V. carteri*. In addition, we demonstrate the expression of a heterologous reporter gene, the codon-optimized luciferase gene of *G. princeps*, driven by promoters of *C. reinhardtii* or *V. carteri*.

## Results

### Antibiotic tolerance of wild-type *Eudorina* algae

**Figure 2:**
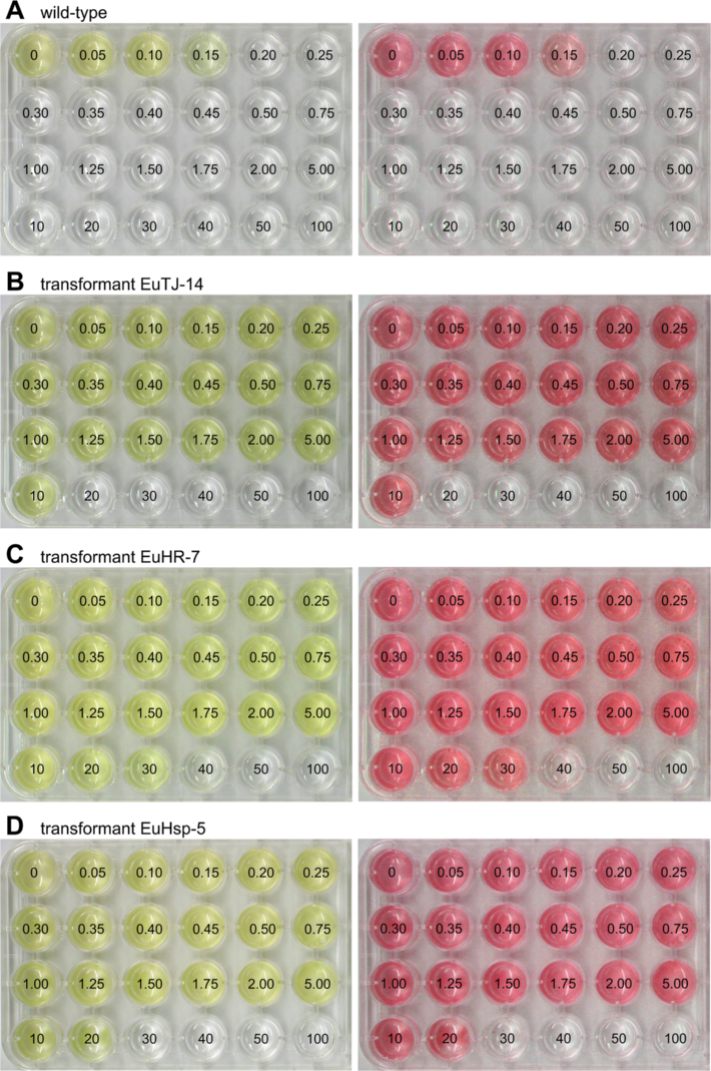
**Analysis of paromomycin resistance in wild**-**type and transgenic *E. elegans *strains.** For analysis of antibiotic resistance, identical quantities of wild-type or transgenic *Eudorina* cells were exposed to increasing concentrations of paromomycin, and incubated for 10 days. Numbers refer to the concentration of paromomycin [μg/ml] utilized. Natural color (left) and red-shifted, false-color images (right) are shown. (**A**) Wild-type *E*. *elegans* strain UTEX 1193 used as a reference control. (**B**-**D**) Transgenic *E*. *elegans* strains co-transformed with pPmr3, the selectable marker plasmid, in addition to a second, non-selectable reporter gene plasmid. (**B**) Transformant EuTJ-14 was co-transformed with the plasmids pPmr3 and pPsaD-GLuc. (**C**) Transformant EuHR-7 was co-transformed with the plasmids pPmr3 and pHRLucP. (**D**) Transformant EuHsp-5 was co-transformed with the plasmids pPmr3 and pHsp70A-GLuc.

The lowest concentration of the antibiotic paromomycin that still kills all wild-type *Eudorina* cells was determined to allow for selection of transformants with potentially weak transgene-mediated resistance. To investigate the paromomycin tolerance of the wild-type *E. elegans* strain, identical numbers of cells were exposed to increasing concentrations of paromomycin, incubated for 10 days, and screened for living (green) or dead (white) cells. A concentration of 0.20 μg paromomycin/ml or higher led to 100% cell death (Figure 2A, left panel). Processing of large-scale screenings of culture plates was facilitated by creating red-shifted, false-color images from standard photographs of the plates; this conversion allows for an objective and rapid discrimination between wells containing living cells and wells containing dead cells (Figure 2A, right panel).

### Transformation experiments using an *aph*VIII-based selectable marker

**Figure 3:**
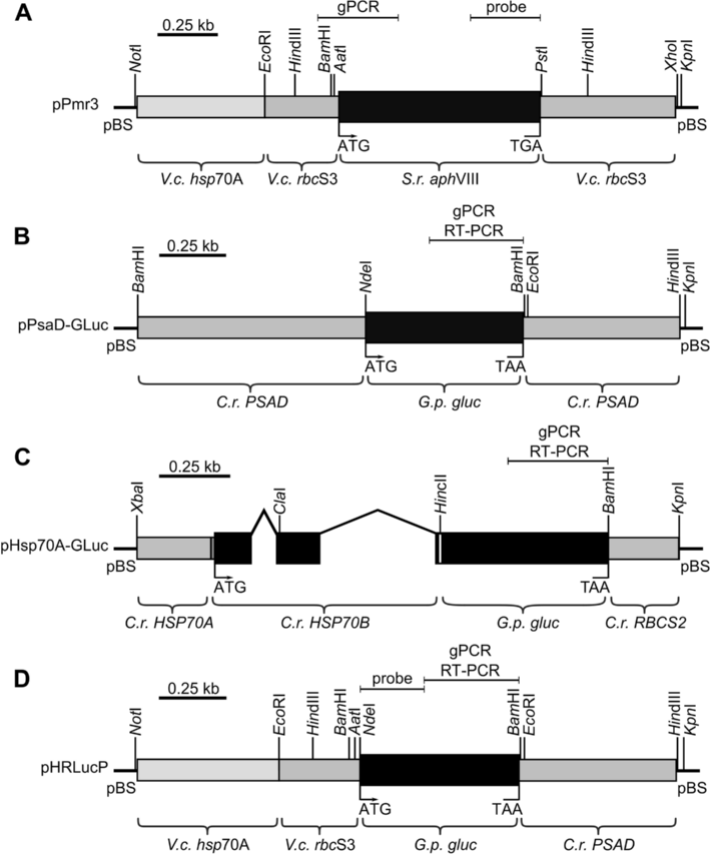
**Schematic diagram of the selectable marker plasmid and co**-**transformed,non-selectable plasmids.** (**A**) The chimeric selectable marker plasmid pPmr3. (**B**-**D**) Co-transformed, non-selectable plasmids containing reporter genes: (**B**) pPsaD-GLuc, (**C**) pHsp70A-GLuc, and (**D**) pHRLucP. The angled lines in (**C**) indicate introns. (**A**-**D**) Amplified genomic (gPCR) or RT-PCR fragments as well as probes used for Southern blots are indicated. *V*.*c*., *Volvox carteri*; *C*.*r*., *Chlamydomonas reinhardtii*; *S*.*r*., *Streptomyces rimosus*; *G*.*p*., *Gaussia princeps*; *gluc*, luciferase gene, pBS, pBluescript II vector.

For the transformation of *E. elegans*, a chimeric, *aph*VIII-based plasmid (pPmr3) [67] was used as a selectable marker. The pPmr3 plasmid contains the coding sequence of the original *S. rimosus aph*VIII gene, a 5^′^-flanking sequence that includes an artificial tandem promoter from the *hsp*70A and *rbc*S3 genes of *V. carteri*, and a 3^′^-flanking sequence derived from the *rbc*S3 gene of *V. carteri* (Figure 3A).

Transformation of wild-type *E. elegans* cells was performed using a Biolistic® PDS-1000/He particle gun and DNA-coated gold microprojectiles [17]. For the transformation, approximately 3 × 10^5^ logarithmically growing *Eudorina* algae were harvested by centrifugation; this corresponds to approximately 9 × 10^6^ target cells. The algae were immobilized on a membrane filter, and excess liquid was removed. During development of the transformation protocol, transformation parameters were modified until paromomycin-resistant transformants were obtained (see below). The protocol was then modified to increase the number of transformants. The most successful combination of parameters for transformation of *E. elegans* is summarized in Additional file 1. The transformation protocol included the use of microprojectiles with a diameter of 0.6 μm, which were coated with 5 μg of the selectable marker plasmid pPmr3 and, where applicable (see below), 5 μg of a non-selectable, co-transformed plasmid. The burst pressure of the rupture discs was 1,100 psi, and the distance between the rupture disc and the macrocarrier was adjusted to 8 mm. The distance between the macrocarrier and the stopping screen was set to 7 mm, and the distance between the stopping screen and target cells was adjusted to 8 cm. After a recovery phase of 24 h in standard liquid medium, the antibiotic resistant transformants were selected by the addition of paromomycin (2 μg/ml). It should be noted that all transformants exhibited the same morphological phenotype as their wild-type parental strain. The detailed transformation protocol is provided in the Methods section. After establishing the transformation protocol, each transformation experiment yielded approximately 3–12 antibiotic resistant clones.

### Antibiotic resistance of transformants

Transformants from five independent transformation experiments (i.e., 33 clones) were tested for their maximal antibiotic resistance. The paromomycin resistance of the clones varied, with a wide range from 5 to 50 μg/ml (Figure 2B–D; also see Additional file 2); however, there was little variation for a given clone. The transformants with the lowest resistance to paromomycin treatment were capable of surviving in 33-fold higher concentrations of paromomycin than parental wild-type strains, which tolerated only up to 0.15 μg paromomycin/ml. The transformants with the most robust resistance tolerated concentrations of antibiotic up to 333-fold higher than the wild-type strains. The variation in resistance between different clones could reflect position effects on expression of the transgene due to random integration or could reveal gene-dosage effects caused by variable number of copies integrated into the genome.

### Stable integration of plasmid DNA into the genome of transformants

**Figure 4:**
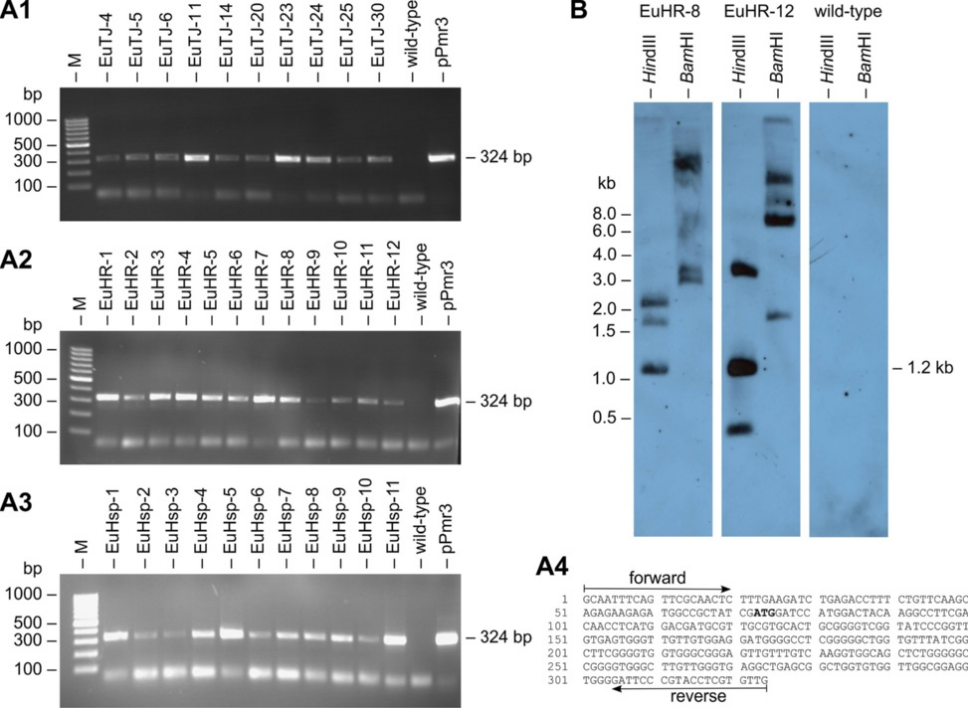
**Detection of the *aph *VIII gene in transformants.** (**A1**-**A3**) Paromomycin-resistant transformants and the parental wild-type strain were analyzed for presence of the *aph*VIII gene in the genome by genomic PCR. The expected size of the *aph*VIII fragment produced in transformants was 324 bp (Figure 3A). The rightmost lane (pPmr3) shows a positive control using pPmr3 plasmid DNA as the template. Lane M refers to the molecular weight marker. (**A4**) Sequence obtained from the amplified and cloned *aph*VIII fragments. The positions of the primers and the start codon (bold) are indicated. (**B**) Southern blot analysis of *Hin*dIII- or *Bam*HI-digested genomic DNA of two randomly chosen transformants, EuHR-8 and EuHR-12, and from the parental wild-type strain. The blot was probed using a 282-bp fragment of the *aph*VIII coding region (Figure 3A).

The stable integration of the selectable marker gene (encoded on the pPmr3 plasmid) into the genome of the 33 clones from five independent transformation experiments was verified by PCR using *aph*VIII-specific oligonucleotide primers (Figure 4A4) and genomic DNA as the template. A PCR fragment of the expected size was obtained from all paromomycin-resistant clones; as expected, the parental wild-type strain failed to produce this fragment (Figure 4A1–A3). The PCR fragment was verified by sequencing (Figure 4A4).

In addition, genomic DNA from transformants and from the parental wild-type strain was analyzed by Southern blot for integration of the *aph*VIII gene into the genome. A fragment of the *aph*VIII coding region (Figure 3A) was used as a probe for the Southern blot. Hybridization signals were detectable only in transformants and not for the parental strain. A Southern blot result for two randomly chosen transformants and the parental wild-type is shown in Figure 4B. Based on the sequence of pPmr3, a hybridization signal of 1.2 kb was expected following digestion with *Hin*dIII, and this fragment was observed following the digestion of transformant genomic DNA. At least two additional bands were detectable in *Hin*dIII-digested samples, which could indicate additional incomplete or fragmented integration of the selectable marker plasmid (Figure 4B). The pPmr3 plasmid contained only a single *Bam*HI site. Therefore, the second *Bam*HI site of the *Bam*HI fragments must be located within the genomic sequence flanking the integrated plasmid, and detection of more than one *Bam*HI hybridization signal indicates multiple integration events in different areas of the genome. Up to five signals were detected in *Bam*HI-digested genomic DNA of transformants, which indicates that up to five integration events occurred (Figure 4B). However, several of these pPmr3 fragments may contain an incomplete selectable marker gene as indicated by the *Hin*dIII digests. The antibiotic resistance of transformants demonstrates that at least one copy of the selectable marker gene is complete. The Southern blot experiments indicate that particle gun transformation of *E. elegans* often leads to more than one integration event, but the number of integration events is quite low (5 or fewer events).

### Calculation of transformation frequency

The frequency of transformation was calculated as the ratio between the number of transformants that exhibited both antibiotic resistance and the correct *aph*VIII fragment following PCR and the total number of cells used for each transformation experiment (9 × 10^6^ cells). Based on 5 independent transformation experiments, the transformation frequency was calculated as 3.7 × 10^-7^ (± 14%) transformants per cell. Assuming that all of the *E. elegans* colonies used for transformation contained 32 cells, the calculated transformation frequency corresponds to 1.2 × 10^-5^ (± 14%) transformants per organism.

### Stable co-transformation with non-selectable reporter gene plasmids

Construction of transformation vectors with selectable markers and non-selectable genes of interest in the same plasmid is frequently hampered by the large size of the final plasmid and by inappropriate positions of required restriction sites. Therefore, selectable markers and genes of interest have been frequently introduced on different plasmids by co-transformation of volvocine species [17,46,62,63,67,69,75,77,83].

To investigate the efficiency of co-transformation in *Eudorina*, we used pPmr3 as a selectable marker plasmid, which contains the *aph*VIII gene, in conjunction with one of three different non-selectable reporter gene plasmids. The non-selectable reporter plasmids contain the *gluc* reporter gene from the *G. princeps* copepod, which was previously engineered to match the codon usage of *C. reinhardtii*[82], and different heterologous flanking sequences including promoters from *C. reinhardtii* and *V. carteri*. The pPsaD-GLuc plasmid contains the *gluc* gene, a 5^′^-flanking sequence of the *C. reinhardtii PSAD* gene (including the *PSAD* promoter and the 5^′^-UTR), and a 3^′^-flanking sequence from the *PSAD* gene (including the 3^′^-UTR) [82] (Figure 3B). The pHsp70A-GLuc plasmid contains a 5^′^-flanking sequence from the *C. reinhardtii HSP70A* gene, which includes the *HSP70A* promoter and the 5^′^-UTR [82] (Figure 3C). This sequence is fused to a genomic fragment of the *C. reinhardtii HSP70B* gene, which contains the first two exons and a small part of the third exon of *HSP70B*. The fragment also contains the two original introns of the *HSP70B* gene, and the exons of the *HSP70B* fragment encode the chloroplast transit peptide of HSP70B [84]. The *gluc* gene is fused downstream of the *HSP70B* sequence, and *gluc* is followed by the 3^′^-flanking sequence of the *C. reinhardtii RBCS2* gene, which includes the 3^′^-UTR (Figure 3C). The pHRLucP plasmid contains the *gluc* gene flanked by a *V. carteri hsp*70A/*rbc*S3 hybrid sequence on its 5^′^-side; this sequence includes the *hsp*70A/*rbc*S3 tandem promoter and a 5^′^-UTR. On the 3^′^-side of the *gluc* gene, the 3^′^-flanking sequence of the *C. reinhardtii PSAD* gene follows, which includes the 3^′^-UTR (Figure 3D). Both the *C. reinhardtii HSP70A* promoter in the pHsp70A-GLuc plasmid and the *V. carteri hsp*70A part of the tandem promoter in the pHRLucP plasmid are known to increase transcription following a period of elevated temperature.

**Figure 5:**
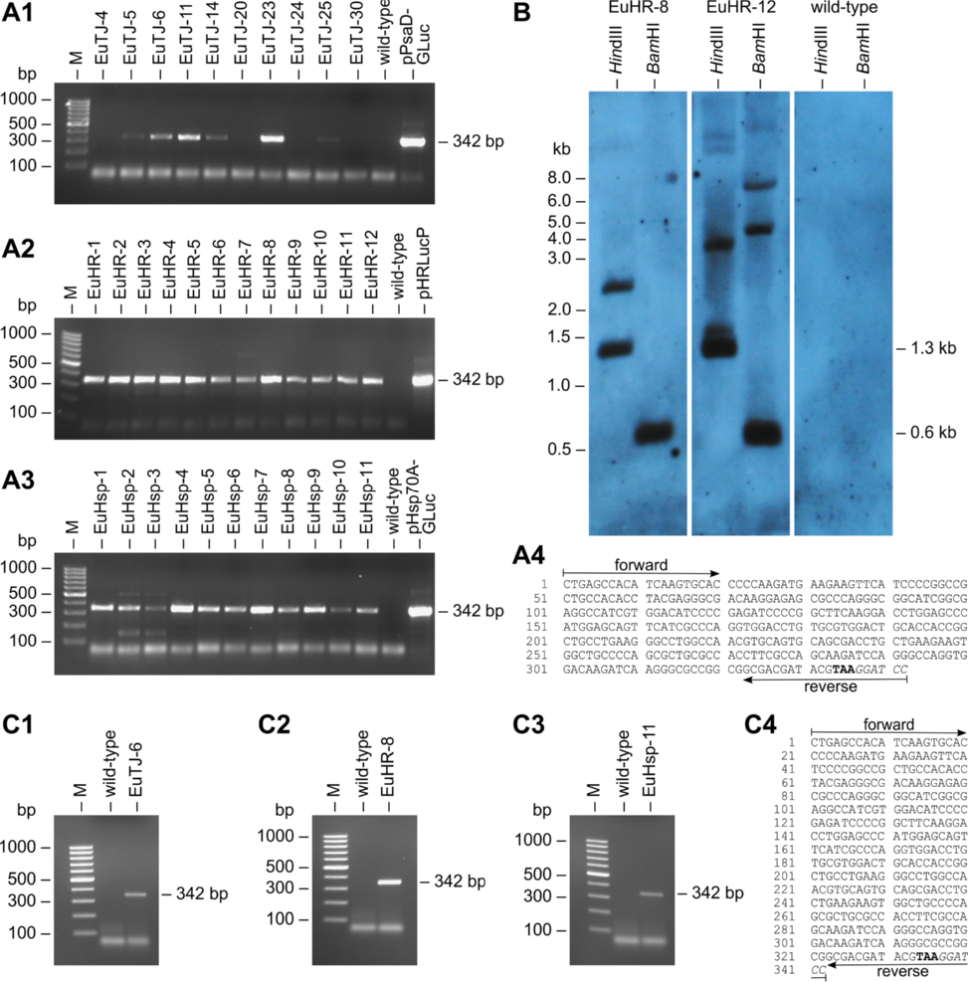
**Demonstration of co-transformation and transcription of heterologous genes.** (**A1**-**A3**) Paromomycin-resistant transformants were analyzed for the presence of the co-transformed, non-selectable DNA in the genome. PCRs were conducted using genomic DNA from transformants co-transformed with the pPsaD-GLuc (**A1**), pHRLucP (**A2**), or pHsp70A-GLuc (**A3**) plasmids as template. The parental wild-type strain was analyzed as a control. Primers were specific for the heterologous *gluc* gene, and a 342-bp PCR fragment (Figure 3B–D) was expected in co-transformants. The rightmost lane shows a positive control using DNA of the co-transformed plasmid as the template. Lane M refers to the molecular weight marker. (**A4**) Sequence obtained from the amplified and cloned *gluc* fragments. Primer positions, the stop codon (bold), and a *Bam*HI restriction site (italics) are indicated. (**B**) Southern blot analysis of *Hin*dIII- or *Bam*HI-digested genomic DNA from transformants EuHR-8 and EuHR-12, which were produced using the pPmr3 and pHRLucP plasmids, and from the parental wild-type strain. The blot was probed using a 258-bp fragment of the *gluc* gene (Figure 3D). (**C1**-**C3**) Transcription analysis by RT-PCR. RNA from paromomycin-resistant transformants co-bombarded with the non-selectable plasmids pPsaD-GLuc (**C1**), pHRLucP (**C2**), or pHsp70A-GLuc (**C3**) was reverse transcribed, and products were amplified by PCR using *gluc*-specific primers. Co-transformants were expected to yield a 342-bp cDNA fragment of the *gluc* gene (Figure 3B–D). The parental wild-type strain was used as a control. (**C4**) Sequence obtained from the cloned *gluc* cDNA fragments. Primer positions, the stop codon (bold), and a *Bam*HI restriction site (italics) are indicated. Lane M refers to the molecular weight marker.

The wild-type *E. elegans* strain was co-bombarded with the selectable marker plasmid (pPmr3) and with one of the non-selectable plasmids (pPsaD-GLuc, pHsp70A-GLuc, or pHRLucP) (Figure 3B–D). The presence of the co-bombarded plasmids in the genome of the transformants was verified by PCR using *gluc*-specific oligonucleotide primers and genomic DNA as the template. The expected PCR fragment of *gluc* (Figure 3B–D) was detected in almost all of the paromomycin-resistant transformants (Figure 5A1–A3), and the identity of the PCR fragment was verified by sequence analysis (Figure 5A4). In detail, five co-transformation experiments with pPmr3 and the non-selectable plasmids pPsaD-GLuc, pHsp70A-GLuc, and pHRLucP yielded 10, 11, and 12 paromomycin-resistant transformants, respectively. Of these, 6 (pPsaD-GLuc), 11 (pHsp70A-GLuc), and 12 (pHRLucP) transformants contained the non-selectable reporter genes. Based on these results, the co-transformation rate was calculated to be 50 - 100%.

Moreover, the stable integration of the *gluc* gene into the genome of *Eudorina* as well as the copy number of the integrations was investigated by Southern blot analyses using a *gluc*-specific probe. The Southern blot results for two randomly chosen transformants are shown in Figure 5B. For hybridization experiments with *Hin*dIII-digested genomic DNA from co-transformants, a DNA fragment of 1328 bp was expected, and fragments were detected in co-transformants. Likewise, hybridization to *BamH*I-digested DNA was expected to produce a 603 bp fragment, and this fragment was detected in the genomic DNA of transformants (Figure 5B). In addition, hybridization signals of various sizes were detected in several co-transformants (e.g., EuHR-12 in Figure 5B), indicating additional integration of reporter gene fragments. However, the number of these fragment integrations was below five.

### Transcription of co-transformed, non-selectable reporter genes

Total RNA of the transformants and *gluc*-specific oligonucleotide primers were used for RT-PCR to investigate the transcription of the heterologous *gluc* gene under the control of heterologous promoters from *V. carteri* and *C. reinhardtii* in transgenic in *E. elegans* strains. A 342-bp cDNA fragment of *gluc* was expected following RT-PCR (Figure 3B–D). The *gluc* cDNA fragments were obtained from transformants that contained the chimeric *gluc* genes following co-transformation with the pPsaD-GLuc, pHRLucP, and pHsp70A-GLuc plasmids (Figure 5C1–C3). The identity of the RT-PCR fragments was verified by sequence analysis (Figure 5C4). As expected, wild-type parental strains did not produce this fragment (Figure 5C1–C3). Thus, the chimeric, non-selectable *gluc* reporter gene was successfully transcribed in *E. elegans* transformants under the control of various heterologous promoters from *V. carteri* and *C. reinhardtii*.

### Analysis of heterologous protein expression in co-transformants

**Figure 6:**
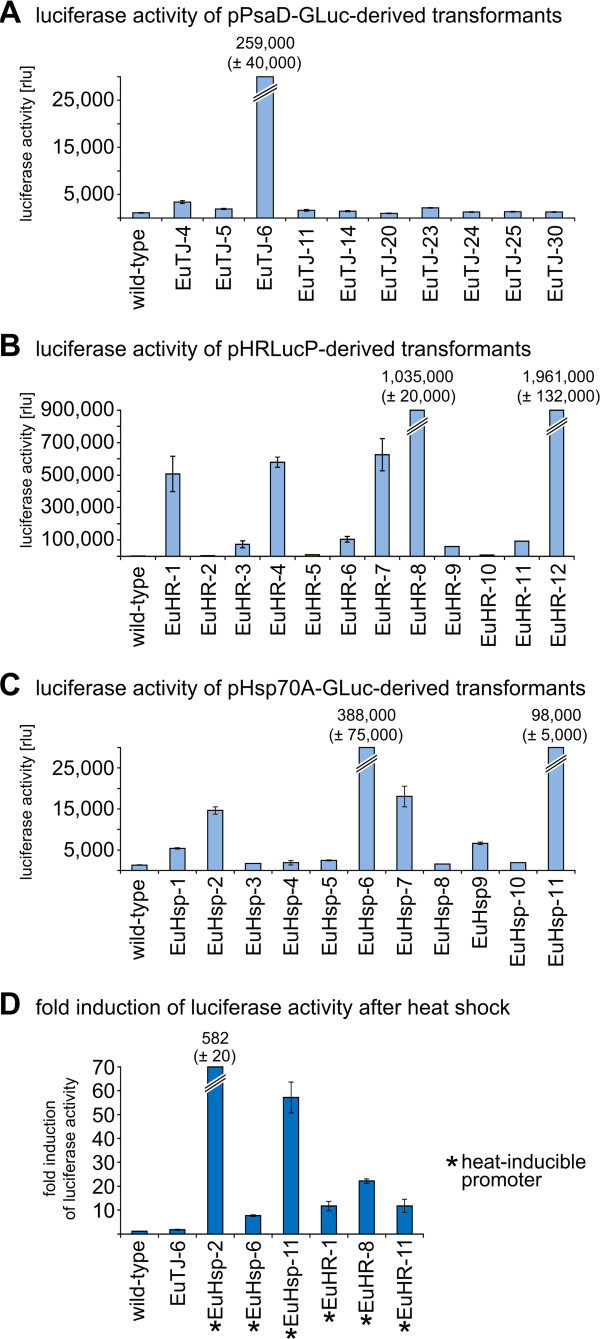
**Quantification and inducibility of luciferase activity in transformants expressing the luciferase gene.** Luciferase activity in transformants was assayed using a luminometer. The bars represent the mean of three independent experiments. The standard deviation is indicated. (**A**) Luciferase activity of pPsaD-GLuc-derived transformants (EuTJ…) compared to the wild-type strain. (**B**) Luciferase activity of pHRLucP-derived transformants (EuHR…) compared to the wild-type strain. (**C**) Luciferase activity of pHsp70A-GLuc-derived transformants (EuHsp…) compared to the wild-type strain. (**D**) Fold induction of luciferase activity in heat-shocked transformants (42°C) compared to non-heat-shocked transformants. In pPsaD-GLuc-derived transformants (EuTJ…), the luciferase gene is driven by the *Chlamydomonas reinhardtii psaD* promoter, and in pHRLucP-derived transformants (EuHR…), the luciferase is driven by the *Volvox carteri hsp*70A/*rbc*S3 tandem promoter. For pHsp70A-GLuc-derived transformants (EuHsp…), the luciferase gene is driven by the *Chlamydomonas reinhardtii HSP70A* promoter. Transformants with a *gluc* gene driven by a heat-inducible promoter are indicated (*). The parental wild-type strain was analyzed as a control. Transformants and wild-type colonies were subjected to a temperature shift from 27°C to 42°C for 1 h. After a 15 min recovery phase at 27°C, cells were lysed, and luciferase activity was assayed. As a reference control, non-heat-shocked transformants were analyzed in an identical fashion.

All transformants that contained the co-transformed *gluc*-construct in their genomes were analyzed for functional expression of the luciferase enzyme. The parental wild-type strain was used as a negative control. A luminometer was used to quantify luciferase activity following cultivation of the strains under standard conditions, and luminescence values were recorded as relative light units (rlu) (Figure 6A–C).

Among those that were co-transformed with the pPsaD-GLuc plasmid, only a single co-transformant, EuTJ-6, exhibited strong luciferase activity (259,000 ± 40,000 rlu) (Figure 6A). The parental wild-type strain only produced a very low background value (1,100 ± 30 rlu).

Contrary to the result with pPsaD-GLuc co-transformants, several pHRLucP co-transformants exhibited strong luciferase activity (Figure 6B). Like in the previous experiment, the background value of the wild-type strain was very low (1,100 ± 50 rlu). It should be noted that the luciferase activity levels in co-transformants were quite variable. For example, the enzymatic activity of co-transformants EuHR-2 (4000 ± 90 rlu) and EuHR-12 (1,961,000 ± 132,000 rlu) differed by a factor of nearly 500 (Figure 6B). The luciferase activity in the EuHR-12 strain was the highest luciferase activity detected among all transformants generated using the *gluc* gene (when cultured under standard conditions).

Also among the co-transformants produced using the pHsp70A-GLuc plasmid, several strains exhibited strong luciferase activity (Figure 6C). The lowest activity was detected in co-transformant EuHsp-1 (5,400 ± 170 rlu), and the highest activity was observed in EuHsp-6 (388,000 ± 75,000 rlu). The enzymatic activity of pHsp70A-GLuc co-transformants differed by a factor of about 70.

In all luciferase-expressing co-transformants, the enzymatic activity was stable. Thus, even the extreme variability in luciferase activity between different transformants was permanent.

### Inducibility of heterologous protein expression

Inducible promoters are useful tools in gene technology, and the heat shock promoters used here are induced at elevated temperatures, at least in the species where they originate. To investigate the inducibility of the *V. carteri hsp70A*/*rbcS3* promoter of the pHRLucP plasmid (Figure 3D) and the *C. reinhardtii HSP70A* promoter of the pHsp70A-GLuc plasmid (Figure 3C), which drive the *gluc* gene in transgenic *E. elegans*, the corresponding transformants were exposed to elevated temperatures, and the luciferase activity was measured. Thus, cultures were incubated at different temperatures ranging from 27°C to 57°C (for 1 h). Cultures were then allowed to recover at 27°C (for 15 min), harvested by centrifugation, and disrupted by sonification, and finally, the luciferase activity was measured in a luminometer. Induction factors were calculated by comparing luminescence values with untreated, reference cultures of the same strain; reference cultures were kept at standard conditions (27°C). The strongest luciferase activity induction was achieved in cultures that were incubated at 42°C (see Additional file 3). The highest induction factor obtained at 42°C was 582 (± 20) and was observed in a strain generated by co-transformation with the pHsp70A-GLuc plasmid (EuHsp-2, Figure 6D). Transformants generated by co-transformation with pHRLucP revealed an up to 22 (± 1)-fold induction (EuHR-8, Figure 6D). The wild-type strain and the transformants generated with the pPsaD-GLuc plasmid, which does not contain a heat-inducible promoter, exhibited no induction of luciferase activity, as expected (wild-type and EuTJ6, Figure 6D). Thus, both the *HSP70A*-promoter of *C. reinhardtii* and the *hsp*70A promoter of *V. carteri* are heat-inducible even when utilized in *E. elegans*.

**Figure 7:**
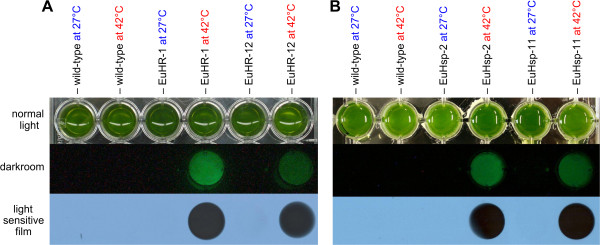
**Visualization of inducible luciferase activity.** Luciferase assay results for parental wild-type colonies and for several transformants with or without heat shock. (**A**) Transformants EuHR-1 and EuHR-12, generated with pHRLucP as the co-bombarded plasmid. (**B**) Transformants EuHsp-2 and EuHsp-11, generated with pHsp70A-GLuc as the co-bombarded plasmid. Algae cultures were divided into two aliquots; one aliquot was subjected to a heat shock at 42°C for 1 h, and the other aliquot was maintained at 27°C. Upper row: standard photo showing the assay setup. Middle row: photo without extraneous light in the darkroom following the addition of the coelenterazine substrate. Lower row: exposure to a light-sensitive film.

We investigated the detection of light in heat-induced transformants using a digital camera and a light-sensitive film in order to optimize the assay for high-throughput setups. Logarithmically growing cultures were divided into two cultures, and one was subjected to temperature of 42°C. The other was maintained at 27°C as described above. Following cell lysis, the transformants were assayed for luciferase activity in 24-well tissue culture plates (Figure 7A, B). The bioluminescence of heat-shocked transformants was not only strong enough to generate a signal on light-sensitive film (Figure 7A, B), but the luminescence was also visible to the naked eye in the dark room and detectable by a standard digital camera (Figure 7A, B).

### In-gel activity assays

The utility of a heterologous reporter gene in *E. elegans* would increase if the gene product could be easily detected even in raw cell extracts after separation on standard SDS-PAGE, which could allow for simple detection and size determination of fusion proteins that contain the Gluc reporter. For the expression product of the *gluc* gene encoded by the pHRLucP plasmid, an expected molecular weight of 21 kDa was calculated based on the amino acid sequence of the Gluc protein. For the HSP70B/Gluc fusion protein encoded by pHsp70A-GLuc, which contains the *HSP70B* chloroplast transit peptide, an expected molecular mass of 31 kDa was calculated.

**Figure 8:**
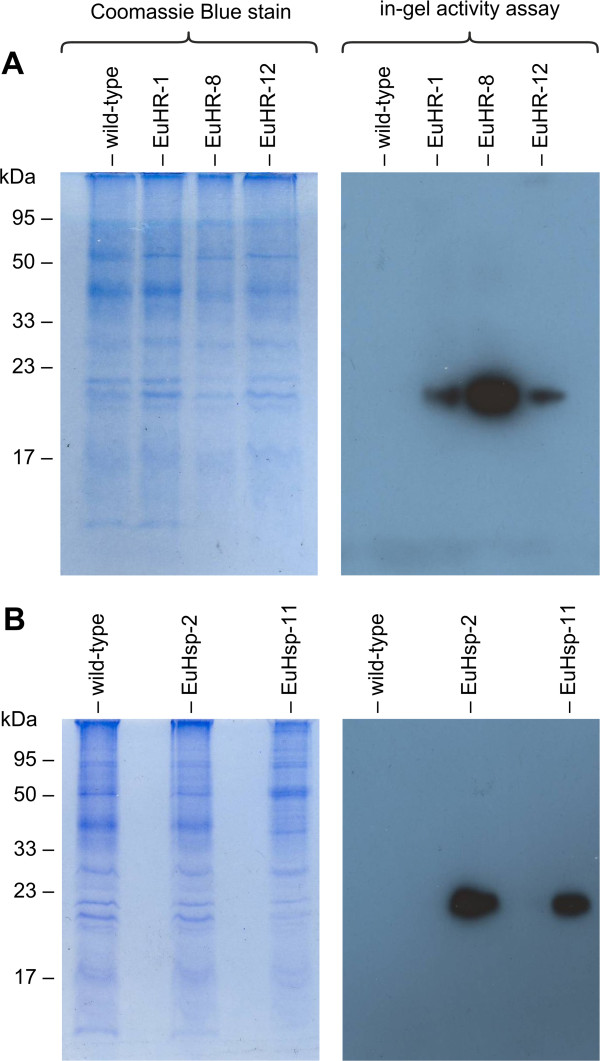
**Detection of luciferase activity by in-gel activity assays.** Cell extracts of heat-shocked transformants were subjected to SDS-PAGE. Subsequently, in-gel renaturation was performed, the coelenterazine-substrate was added, and the gel was exposed to a light-sensitive film (right panels). As a loading control, the same extracts were also stained with Coomassie Blue following SDS-PAGE (left panels). (**A**) Analysis of transformants EuHR-1, EuHR-8, and EuHR-12, which were generated with pHRLucP as the co-bombarded plasmid. (**B**) Analysis of transformants EuHsp-2 and EuHsp-11, generated with pHsp70A-GLuc as the co-bombarded plasmid. In each experiment, the parental wild-type strain was analyzed as a control.

Protein extracts of transformants generated by co-transformation with the pHRLucP or pHsp70A-GLuc plasmid were separated by standard SDS-PAGE [85] without using thiol reagents. After electrophoresis, in-gel renaturation of luciferase was achieved in the presence of β-cyclodextrin, a cyclic oligosaccharide [86]. The renatured enzyme was assayed in the gel by addition of the substrate and detection of bioluminescence on light-sensitive film. In these in-gel-luciferase activity assays, co-transformants generated with the pHRLucP plasmid exhibited strong luciferase signals of the expected size (~21 kDa) (Figure 8A). Co-transformants generated with pHsp70A-GLuc also showed strong signals of approximately 21 kDa (Figure 8B), indicating that the heterologous HSP70B chloroplast transit peptide of *C. reinhardtii* is cleaved in *E. elegans*.

### Long-term stability of DNA integration and gene expression

The long-term stability of transgenes, which have been randomly integrated into the genome, and the perpetuation of their expression are beneficial for any genetically engineered organism.

The stability of the *aph*VIII and *gluc* transgenes within the genomes of transgenic *E. elegans* strains was investigated by re-examining the 33 transformants for resistance to paromomycin and luciferase activity after 12 months without any selective pressure, which reflects 300 generations of cultivation. Even after this period, the resistance of the transformants to paromomycin, which was determined at the highest possible antibiotic concentrations, varied by only approximately ± 5% from the values determined at the initial time-point (see above). Likewise, the luciferase activity in transformants differed from the initial values by only approximately ± 6%. Thus, the integration and expression of the heterologous *aph*VIII and *gluc* genes are stable in *E. elegans*.

## Discussion

Stable nuclear transformation of *E. elegans* is now feasible using a chimeric selectable marker from a heterologous source. Additionally, chimeric reporter gene constructs and constitutive and inducible promoters are provided without using endogenous genes or DNA elements. We also demonstrate that inducible, heterologous promoters can retain their inducibility in the new cellular environment of the target species.

The frequency of transformation for *E. elegans* was estimated to be 3.7 × 10^-7^ transformants per cell. A comparable value of 1.1 – 6.6 × 10^-7^ transformants per cell has been reported for the related *G. pectorale* species [17]. The transformation frequency in *C. reinhardtii* was calculated as 1.3 - 1.9 × 10^-7^ transformants per cell [87], which is similar to the values above. In *V. carteri*, the transformation frequency was calculated per organism and not per cell; this value was reported to be 2.5 × 10^-5^[44]. The authors used a *V. carteri* mutant, which carries a mutation that causes somatic cells to redifferentiate as asexual reproductive cells. If approximately 200 of the 2000–4000 cells composing this mutant actually reproduced, the estimated transformation frequency per cell would be approximately 1.25 × 10^-7^, which is similar to the rates mentioned above. Thus, the reported transformation frequencies for all of the investigated volvocine species appear to be quite similar.

Co-transformation of a selectable marker plasmid and the gene of interest on a separate plasmid is a convenient strategy for dealing with large genes and complex gene constructs [17,46,62,63,67,69,75,77,83]. Moreover, the same selectable marker plasmid can be used for all transformation experiments. The co-transformation rate in *E. elegans* was determined to be 50 - 100%. For *V. carteri*, rates of 10 - 60% [63] or 40 - 80% [44] have been reported. The co-transformation rates for *C. reinhardtii* are 50% [88] or approximately 80% [63], and a co-transformation rate of 30 - 50% [17] has been reported for *G. pectorale*. However, the varying co-transformation rates reported for different volvocine species may not represent species-specific values, but they could be the result of different plasmid sequences, plasmid sizes, or (co-)transformation conditions. Although the co-transformation rates vary over a wide range in volvocine algae (10 – 100%), all rates are quite high, and therefore, co-transformation can be used routinely in all volvocine species. Even the lowest rate of 10% means that one co-transformant with the gene of interest can be identified from only ten antibiotic-resistant transformants, which can be easily detected by PCR. Therefore, transformation of volvocine algae does not require construction of vectors that contain all required genes and regulatory elements on a single plasmid.

The transformation protocol for *E. elegans* described above can now also be used for insertional mutagenesis of the *E. elegans* genome, which has been done in *C. reinhardtii*[89,90]. Insertional mutagenesis is one of the most powerful methods for determining the cellular function of a particular gene. For insertional mutagenesis, the pPmr3 plasmid (Figure 3A) could be used as currently constructed; the plasmid only needs to be linearized (e.g., by digesting with the restriction enzymes *Not*I or *Kpn*I). Fortunately, the number of integrated copies of the transforming plasmid was low in our *E. elegans* transformation experiments, and these values were comparable to the copy numbers reported for insertional mutagenesis studies in *C. reinhardtii*[89]. Insertional loss-of-function mutants can be screened for developmental or physiological phenotypes, and the disrupted genes flanking the transforming DNA can be amplified from insertional mutants using thermal asymmetric interlaced (TAIL)-PCR [91], sequenced, and characterized.

## Conclusions

Both heterologous coding regions and promoters from related volvocine species work in *E. elegans* and drive both constitutive and inducible expression of heterologous genes. The availability of a transformation system, constitutive and inducible promoters, selectable markers, and reporter genes now makes extensive engineering of *E. elegans* possible. The results also demonstrate that genetic engineering of a species is possible even without endogenous genes and promoters.

## Methods

### Strains and culture conditions

The wild-type *Eudorina elegans* strain UTEX 1193 was obtained from R. Schmitt (University of Regensburg, Germany). *Eudorina* cultures were maintained in *Volvox* medium (VM) [92] at 27-29°C in an 8 h dark/16 h light (~10,000 lux) cycle. Cultures were grown in 10 ml glass tubes, 50 or 300 ml Erlenmeyer flasks, or 1,000 ml Fernbach flasks. The glass tubes had caps that allow for gas exchange, and Erlenmeyer and Fernbach flasks were aerated via Pasteur pipettes with approximately 50 cm^3^ sterile air/min. Transgenic strains expressing the *aph*VIII gene were grown in VM in the presence of 1 μg paromomycin/ml (paromomycin sulfate, Sigma-Aldrich, St. Louis, MO).

### Transformation vectors

The selectable marker pPmr3 plasmid (Figure 3A) is 5.1 kb in size and contains 0.77 kb of upstream sequence, which includes a *V. carteri hsp*70A-*rbc*S3 tandem promoter (0.5 kb of *V. carteri hsp*70A and 0.27 kb of *V. carteri rbc*S3 sequences), the 0.8 kb coding region of the *S. rimosus aph*VIII gene, the 3^′^-UTR from the *V. carteri rbc*S3 gene (0.53 kb of downstream sequence), and the pBluescript vector backbone [67] [GenBank: AY429514]. The pPsaD-GLuc plasmid (Figure 3B) is 5.0 kb in size and contains 0.8 kb of upstream sequence, which includes the *C. reinhardtii PSAD* promoter, the 0.57 kb coding region of the *G. princeps gluc* gene (optimized for the codon usage of *C. reinhardtii*), the 3^′^-UTR of the *C. reinhardtii PSAD* gene (0.56 kb of downstream sequence), and the pBluescript vector backbone [73,82] [GenBank: EU372000, AF335592]. The pHsp70A-Gluc plasmid (Figure 3C) is 4.9 kb in size and contains 0.26 kb of upstream sequence, which includes the *C*. *reinhardtii HSP70A* promoter, a 0.8 kb fragment of the *C*. *reinhardtii HSP70B* genomic sequence (including two introns) fused to a 0.57 kb coding region of the codon-optimized *G*. *princeps gluc* gene, the 3^′^-UTR of the *C*. *reinhardtii RBCS2* gene (0.22 kb of downstream sequence), and the pBluescript vector backbone [82]. For construction of the pHRLucP plasmid, a 1.2 kb DNA fragment containing the codon-optimized *G*. *princeps gluc* gene (0.57 kb) and the 3^′^-UTR of the *C*. *reinhardtii PSAD* gene (0.56 kb of downstream sequence) was amplified by PCR using the following primers: 5^′^-TAGGCCTAACAAGCCCAT*ATG*GTC (ON15486) and 5^′^-GGAAACAGCTATGACCATG (M13-reverse); the pPsaD-GLuc plasmid [82] was used as the template. Primer ON15486 contains an artificial *Aat*I site (restriction site underlined; start codon in italics). The PCR fragment was cloned into the pGEM-T easy vector (Promega, Madison, WI) and cut out with *Aat*I and *Kpn*I; the latter restriction site comes from the cloning site of the vector. The 1.2 kb *Aat*I/*Kpn*I fragment was ligated with a 3.8 kb *Aat*I/*Kpn*I fragment from the pPmr3 vector, which contains the *V*. *carteri hsp*70A-*rbc*S3 tandem promoter (0.77 kb) and the pBluescript vector backbone. The final plasmid, pHRLucP (Figure 3D), is 5.0 kb in size and contains 0.77 kb of upstream sequence containing the *V*. *carteri hsp*70A-*rbc*S3 tandem promoter, the 0.57 kb coding region of the *G*. *princeps gluc* gene, the 3^′^-UTR of the *C*. *reinhardtii PSAD* gene (0.56 kb), and the pBluescript vector backbone.

### Preparation of plasmid DNA

Plasmid DNA was purified using the Nucleospin® Plasmid Kit according to the manufacturer’s instructions (Macherey-Nagel, Düren, Germany).

### Coating of microprojectiles

Gold microprojectiles (0.6 μm in diameter, Bio-Rad, Hercules, CA) were coated with the required plasmids for biolistic transformation [17]. For the transformation, approximately 3 mg of the gold microprojectiles in 50 μl of H_2_O was quickly mixed with 5 μg of the circular selectable marker plasmid DNA (concentration >0.5 μg/μl), 5 μg of the circular co-bombarded plasmid (concentration >0.5 μg/μl), 40 μl of CaCl_2_ (2.5 M), and 20 μl of spermidine (0.1 M, Sigma-Aldrich) [17]. Mixing was sustained for 30 min at 4°C, after which 200 μl of ethanol (at room temperature) was added and the suspension centrifuged for 2–3 s at ~5,000 g. The pellet was washed three times with 100 μl of ethanol (at −20°C) and centrifuged for 2–3 s at ~5,000 g. Finally, the DNA-coated microprojectiles were resuspended in 60 μl of ethanol and kept at 4°C; these microprojectiles were used within 3 h of preparation.

### Determination of cell concentration

In *E*. *elegans*, the number of cells per colony varies significantly not only among individuals cultured under different conditions but also among individuals cultured under the same conditions. Therefore, we report the concentration as cells/ml rather than colonies/ml. The concentration of cells was determined using a hemacytometer with Neubauer ruling.

### Stable nuclear transformation by particle bombardment

A logarithmically growing *E*. *elegans* culture (150 ml) at a cell concentration of ~6 × 10^4^ cells/ml was harvested by centrifugation (800 g, 5 min, swing-out rotor) and resuspended in 6 ml of VM. One milliliter of the suspension was evenly spread on a cellulose acetate membrane filter with a pore size of 1.2 μm and a diameter 47 mm (Whatman, London, UK), and the filter was placed on top of a stack of absorbent paper to soak up the excess liquid [17]. The stable transformation of *E*. *elegans* was performed using a Biolistic® PDS-1000/He (Bio-Rad) particle gun. One-sixth of the DNA-coated microprojectiles were spread on a macrocarrier (Bio-Rad), which was placed in a macrocarrier holder (Bio-Rad). In the most successful combination of parameters (see Additional file 1), the distance between the macrocarrier and stopping screen (Bio-Rad) was adjusted to 7 mm and the Helium pressure was set to 1,100 psi (as defined by rupture disks with the corresponding burst pressure). Finally, the distance between rupture disk and macrocarrier was set to 8 mm. The membrane filter with its layer of *E*. *elegans* cells was positioned in the bombardment chamber, and the distance between the target cells and the stopping screen was adjusted to 8 cm. The chamber was partly evacuated to 27 inches of Hg [17]. After the bombardment step, the colonies were washed off of the membrane filter with VM. The procedure was repeated five times, and the algae from six rounds of bombardment were pooled and then evenly distributed among twelve 50 ml Erlenmeyer flasks containing a volume of ~40 ml of VM. The bombarded algae were incubated under standard conditions for 24 h, and then 2 μg of paromomycin/ml was added. The large fraction of non-transformed cells died within 48 h, which resulted in a clarification of the medium. After 9–12 days of incubation in the presence of antibiotic, re-greening of flasks indicated the initial presence and reproduction of at least one paromomycin-resistant *E*. *elegans* cell, which led to a population of transformants. No more than one transformant per flask was analyzed [17].

### Re-isolation of transformants

Transformants were re-isolated for detailed analyses to ensure uniform genetic conditions [17]. For this purpose, a serial dilution of an exponentially growing *E*. *elegans* culture was performed in a Terasaki plate (Nunc™ MicroWell™ MiniTrays; Thermo Fisher Scientific, Langenselbold, Germany). Each well of the Terasaki plate was filled with 10 μl of VM. A single *E*. *elegans* colony was finally transferred into a standard glass tube with VM, containing 1 μg of paromomycin/ml, and incubated under standard conditions.

### Paromomycin-resistance assay

Transformed or wild-type *E*. *elegans* strains were transferred into the wells of a 24-well cell culture plate (Sarstedt, Nümbrecht, Germany) with a wide range of paromomycin concentrations from 0 to 100 μg/ml (concentrations are given in Figure 2A). At the beginning of the assay, each well contained approximately 1,600 cells, which corresponds to 50–70 colonies, in a total VM volume of 1 ml. After incubation under standard conditions for 10 days, the wells were analyzed for viable green cells/colonies or lysed cells/colonies (white).

### Primer design

Oligonucleotide primers were designed using Oligo 6 (Molecular Biology Insights, Cascade, CO), Primer Express® (Applied Biosystems, Foster City, CA), and DNASIS™ (version 7.00; Hitachi Software Engineering, San Francisco, CA).

### Isolation of genomic DNA

Thirty-five milliliters of a logarithmically growing *E*. *elegans* culture (cell density of 2 × 10^6^ cells/ml) was harvested by centrifugation (3,500 g for 10 min). The resulting pellet had a wet weight of ~80 mg. Isolation of genomic DNA was performed using the DNeasy® Plant Mini Kit (Qiagen, Hilden, Germany). Larger amounts of genomic DNA were prepared by conventional methods [93], using tris-saturated phenol (Roti®-phenol, Roth, Karlsruhe, Germany).

### Genomic PCR

PCR utilizing genomic DNA as a template was conducted in a total volume of 50 μl, which contained approximately 100 ng of genomic DNA, 300 nM of each primer, 0.2 mM dNTPs, 1.5 mM MgCl_2_, and 2.6 units of the Expand High Fidelity enzyme mix in 1x Expand High Fidelity buffer (Expand High Fidelity Plus PCR System, Roche Applied Science, Basel, Switzerland). The PCR reactions were performed on a T3 Thermocycler PCR system (Biometra, Göttingen, Germany) using the following conditions: 40 cycles of 94°C for 20 s, 55°C for 30 s, and 72°C for 45 s, followed by a final extension at 72°C for 10 min. The PCR products were cloned and sequenced.

### Southern blotting

Genomic DNA was fragmented by restriction enzyme digestion, and the fragments were separated on 1% agarose gels, vacuum transferred to nylon membranes (Hybond-N®; Amersham Biosciences, Little Chalfont, UK), and fixed to the membrane by baking at 120°C for 30 min using standard protocols [93]. Fragments of the *S*. *rimosus aph*VIII (282 bp; Figure 3A) or the codon-optimized *G*. *princeps gluc* (258 bp; Figure 3D) coding regions were amplified by PCR (Expand High Fidelity Plus PCR System; Roche Applied Science) and simultaneously labeled using a digoxigenin DNA labeling mix (PCR DIG labeling mix; Roche Applied Science). Pre-hybridization at 52°C, hybridization at 52°C, and washing steps were conducted in standard solutions (Roche Applied Science). Hybridizing bands were detected using an anti-digoxigenin-alkaline phosphatase conjugate (1:7500 dilution) and the chemiluminescent substrate CDP Star® following the manufacturer’s instructions for the chemiluminescence reagent (Roche Applied Science). The treated membranes were exposed to light-sensitive film (Retina XBA; Fotochemische Werke, Berlin, Germany) for 2–15 min.

### Isolation of total RNA

Total RNA was extracted from 100 mg (wet weight) of concentrated, frozen algae using 1 ml of the phenol-based TRI Reagent (Sigma-Aldrich, St. Louis, MO) and 300 μl trichloromethane. RNA quantitation and purity checks were conducted by agarose-formaldehyde gel electrophoresis and by measuring absorption at 260 and 280 nm with an Ultrospec 2100 pro UV/Visible Spectrophotometer (GE Healthcare, Uppsala, Sweden).

### Reverse Transcription (RT)-PCR

Reverse transcription was performed using 1 μg of total RNA and Moloney murine leukemia virus (MMLV) reverse transcriptase lacking ribonuclease H activity (H minus) according to the instructions of the manufacturer (Promega). The subsequent PCR was carried out using the Expand High Fidelity PCR system (Roche Applied Science), a T3 Thermocycler PCR system (Biometra), and the following cycling conditions: 40 cycles of 94°C for 20 s, 55°C for 30 s, and 72°C for 45 s, followed by a final extension step at 72°C for 10 min. The RT-PCR products were cloned and sequenced.

### Optimum temperature for heat stress-induced expression of luciferase

Cultures of *E*. *elegans* transformants that express the *gluc* gene following the genomic integration of the co-transformed plasmids pHRLucP or pHsp70A-GLuc were grown logarithmically at 27°C and divided into aliquots of 10 ml. The aliquots were incubated for 1 h at temperatures ranging from 27°C to 57°C (3°C intervals). After a recovery phase at 27°C for 15 min, the algae were harvested by centrifugation (3,500 g for 5 min), resuspended in 300 μl of assay buffer (0.1 M potassium phosphate (pH 7.6), 0.5 M NaCl, 1 mM EDTA), and disrupted by sonification using a Sonopuls™ HD2070 sonicator (Bandelin Electronic, Berlin, Germany). Luciferase activity was measured at 20°C as described previously [82] using a MiniLumat LB9506 luminometer (Berthold, Bad Wildbad, Germany). The luminescence was recorded in relative light units.

### Luciferase assay

For assays on light-sensitive films and for photo-optical documentation of luciferase activity, *Eudorina* cultures (3–6 × 10^6^ cells/ml) were divided into aliquots of 50 ml. One aliquot was incubated at the optimal temperature for heat stress-induced expression of luciferase (42°C) for 1 h, and another aliquot was incubated at 27°C as a reference control. Subsequently, the cultures were centrifuged (3,500 g for 5 min), resuspended in 850 μl of assay buffer, and disrupted by two 30 s pulses of direct sonication using a Sonopuls™ HD2070 sonicator (Bandelin Electronic) at 70% power. Lysates were then transferred into individual wells of a 24-well cell culture plate (Sarstedt). After addition of 150 μl of 0.05 mM coelenterazine (Fluka, Neu-Ulm, Germany) in assay buffer, the 24-well plate was exposed to a chemiluminescence-sensitive film (Retina XBA; Fotochemische Werke, Berlin, Germany) for 2 h at 20°C [94]. In addition, the luciferase activity of transformant lysates in 24-well cell culture plates was documented photo-optically using a Canon Eos 20D photo camera (Canon, Tokio, Japan) with exposure times of 2 to 30 min. Quantification of luciferase activity was conducted as described previously [82] using a MiniLumat LB9506 luminometer (Berthold).

### In-gel activity assay

Logarithmically growing, heat-stressed cultures of *E*. *elegans* transformants expressing the *gluc* gene were centrifuged (4,000 g for 5 min), resuspended in 20 mM sodium phosphate buffer pH 7.6, and disrupted using a Sonopuls™ HD2070 sonicator (Bandelin Electronic). The lysate was cleared by centrifugation (80,000 g for 45 min) at 4°C, and the protein concentration of the cell extract was determined using the Bio-Rad Protein Assay Dye Reagent (Bio-Rad). Cell extracts containing 20 μg of total protein in a volume of 15 μl were mixed with 15 μl of gel loading buffer without thiol reagents, incubated at 23°C for 5 minutes, centrifuged (16,000 g for 2 min), and loaded (without heating) onto a SDS-polyacrylamide gel (15% separation gel, 5% stacking gel). After electrophoresis at 20 mA for 1.5 h (at 4°C), the gel was rinsed in ultrapure water (Milli-Q, Millipore, Bedford, MA) for 20 s. For in-gel renaturation of luciferase, the gel was incubated twice in renaturation buffer (20 mM sodium phosphate buffer (pH 7.6), 0.5% β-cyclodextrin) with gentle shaking for 15 min each. The cyclic oligosaccharide β-cyclodextrin supports protein refolding following SDS-polyacrylamide gel electrophoresis because it is able to capture SDS [86]. After the renaturation step, the gel was incubated in assay buffer for 15 min with gentle shaking and then transferred to a glass-fiber filter paper with a pore size of 0.45 μm (Macherey-Nagel), which was soaked in assay buffer with 0.05 mM coelenterazine substrate. Then, the gel on the filter paper was covered with a thin transparent plastic wrap (SC Johnson, Racine, WI); a light-sensitive film (Retina XBA; Fotochemische Werke, Berlin, Germany) was placed on top, and slight pressure was applied evenly to the sandwich by a flat weight of 150 g. The gel was exposed to the light-sensitive film for 12 h.

## Abbreviations

*aph*VIII: *S. rimosus* aminoglycoside 3^′^-phosphotransferase VIII gene; GFP: Green fluorescent protein; *gluc*: *G. princeps* luciferase gene; *hsp*70A: Heat shock protein 70A gene; MMLV: Moloney murine leukemia virus; PCR: Polymerase chain reaction; *PSAD*: Abundant protein of photosystem I complex gene; *rbc*S3: Ribulose bisphosphate carboxylase small chain gene 3; rlu: Relative light units; RT: Reverse transcription; SDS: Sodium dodecyl sulfate; UTR: Untranslated region; VM: *Volvox* medium.

## Competing interests

The authors declare that they have no competing interests.

## Authors’ contributions

KL conducted the experiments, analyzed the data, and wrote the first version of the manuscript with advice and guidance from AH. AH (corresponding author) conceived and coordinated the study, critically evaluated the data, and finalized the manuscript. Both authors read and approved the final manuscript.

## Supplementary Material

Additional file 1 **Summary of the optimal combination of the parameters for *E. elegans *transformation.** Click here for file

Additional file 2 Paromomycin resistance in transformants. Click here for file

Additional file 3 Luciferase activity of transformants at increased temperatures. Click here for file
