# Supplementary material for: Stable nuclear transformation of Eudorina elegans
Source: BMC Biotechnol. 2013 Feb 12;13:11. doi: 10.1186/1472-6750-13-11 (PMC3576287; doi:10.1186/1472-6750-13-11)
Supplement: Additional file 1 — Summary of the optimal combination of the parameters for E. elegans transformation. [file 1472-6750-13-11-S1.pdf]

Summary of the optimal combination of the parameters for *E. elegans* transformation

Parameter	Parameter specification
particle bombardment system	biolistic PDS-1000/He particle delivery system
target species	wild-type *Eudorina elegans* strain UTEX 1193
number of target cells	approximately 9 x 10^6 cells
preparation of target cells	immobilized on cellulose acetate membrane filter; almost free of liquid
material of microprojectiles	gold
amount of microprojectiles	3 mg for 6 shots
size of microprojectiles	0.6 µm in diameter
selectable marker	pPmr3 (circular)
amount of selectable marker plasmid	5 µg for 6 shots
amount of co-transformed plasmid	5 µg for 6 shots
coating of microprojectiles	plasmid-DNA/microcarrier/$CaCl_2$/spermidine/EtOH-precipitation
burst pressure of rupture disk	1,100 psi
rupture disk-macrocarrier distance	8 mm
macrocarrier-stopping screen distance	7 mm
stopping screen-target cell distance	8 cm
chamber evacuation	27 inch Hg
cultivation after particle bombardment	in liquid medium
