# Supplementary material for: Stable nuclear transformation of Eudorina elegans
Source: BMC Biotechnol. 2013 Feb 12;13:11. doi: 10.1186/1472-6750-13-11 (PMC3576287; doi:10.1186/1472-6750-13-11)
Supplement: Additional file 2 — Paromomycin resistance in transformants. [file 1472-6750-13-11-S2.pdf]

Paromomycin resistance in transformants*

transformant	resistance up to … µg paromomycin/ml**	transformant	resistance up to … µg paromomycin/ml**	transformant	resistance up to … µg paromomycin/ml**
EuTJ-4	20	EuHR-1	20	EuHsp-1	10
EuTJ-5	5	EuHR-2	20	EuHsp-2	20
EuTJ-6	10	EuHR-3	20	EuHsp-3	50
EuTJ-11	20	EuHR-4	20	EuHsp-4	20
EuTJ-14	10	EuHR-5	20	EuHsp-5	20
EuTJ-20	5	EuHR-6	20	EuHsp-6	20
EuTJ-23	30	EuHR-7	30	EuHsp-7	10
EuTJ-24	20	EuHR-8	20	EuHsp-8	10
EuTJ-25	30	EuHR-9	20	EuHsp-9	20
EuTJ-30	20	EuHR-10	20	EuHsp-10	10
		EuHR-11	20	EuHsp-11	20
		EuHR-12	20		

* the maximal tolerance of the wild-type strain *E. elegans* UTEX 1193 was 0.15 µg paromomycin/ml

** the tested concentrations were 0.00, 0.05, 0.10, 0.15, 0.20, 0.25, 0.30, 0.35, 0.40, 0.45, 0.50, 0.75, 1.00, 1.25, 1.50, 1.75, 2, 5, 10, 20, 30, 40, 50, and 100 µg paromomycin/ml
