# Supplementary material for: Stable nuclear transformation of Eudorina elegans
Source: BMC Biotechnol. 2013 Feb 12;13:11. doi: 10.1186/1472-6750-13-11 (PMC3576287; doi:10.1186/1472-6750-13-11)
Supplement: Additional file 3 — Luciferase activity of transformants at increased temperatures. [file 1472-6750-13-11-S3.pdf]

Luciferase activity of transformants at increased temperatures*

Temperature	Wild-type	EuTJ-6	EuHsp-2	EuHsp-6	EuHsp-11	EuHR-1	EuHR-8	EuHR-11
27°C	1.00 (± 0.00)	1.00 (± 0.00)	1.00 (± 0.01)	1.00 (± 0.00)	1.00 (± 0.00)	1.00 (± 0.01)	1.00 (± 0.01)	1.00 (± 0.00)
30°C	1.02 (± 0.00)	1.05 (± 0.00)	1.17 (± 0.04)	1.02 (± 0.01)	1.07 (± 0.03)	1.06 (± 0.02)	1.12 (± 0.04)	1.10 (± 0.03)
33°C	1.07 (± 0.05)	1.14 (± 0.04)	5.02 (± 0.35)	1.16 (± 0.02)	1.59 (± 0.02)	1.15 (± 0.05)	1.29 (± 0.03)	1.12 (± 0.02)
36°C	1.02 (± 0.03)	1.30 (± 0.02)	18.23 (± 0.31)	1.42 (± 0.02)	1.94 (± 0.08)	1.88 (± 0.06)	2.55 (± 0.14)	1.96 (± 0.42)
39°C	0.99 (± 0.07)	1.23 (± 0.16)	55.95 (± 1.09)	1.79 (± 0.05)	5.82 (± 1.63)	3.21 (± 0.38)	6.94 (± 1.32)	4.34 (± 0.15)
42°C	1.11 (± 0.01)	1.86 (± 0.16)	581.50 (± 20.38)	7.70 (± 0.29)	57.13 (± 6.48)	11.71 (± 1.95)	22.17 (± 0.91)	11.77 (± 2.74)
45°C	1.06 (± 0.04)	0.93 (± 0.07)	1.25 (± 0.09)	0.95 (± 0.01)	3.66 (± 1.15)	0.55 (± 0.08)	0.69 (± 0.14)	0.76 (± 0.03)
48°C	0.83 (± 0.04)	0.02 (± 0.00)	0.25 (± 0.02)	0.02 (± 0.01)	0.07 (± 0.02)	0.01 (± 0.00)	0.02 (± 0.00)	0.19 (± 0.01)
51°C	0.69 (± 0.02)	0.02 (± 0.00)	0.09 (± 0.06)	0.02 (± 0.00)	0.05 (± 0.00)	0.01 (± 0.00)	0.01 (± 0.00)	0.13 (± 0.02)
54°C	0.60 (± 0.04)	0.02 (± 0.01)	0.00 (± 0.02)	0.01 (± 0.00)	0.04 (± 0.01)	0.01 (± 0.00)	0.00 (± 0.00)	0.00 (± 0.00)
57°C	0.61 (± 0.01)	0.02 (± 0.00)	0.00 (± 0.02)	0.01 (± 0.00)	0.03 (± 0.00)	0.01 (± 0.00)	0.00 (± 0.01)	0.00 (± 0.00)

* Cells were incubated for 1 h at the given temperature. After a recovery phase at 27°C for 15 min, cells were lysed, and luciferase activity was measured. Fold induction of luciferase activity in heat-shocked cells was calculated in relation to the non-heat-shocked cells at 27°C.
